# *Candida auris*: A Quick Review on Identification, Current Treatments, and Challenges

**DOI:** 10.3390/ijms22094470

**Published:** 2021-04-25

**Authors:** Lucia Černáková, Maryam Roudbary, Susana Brás, Silva Tafaj, Célia F. Rodrigues

**Affiliations:** 1 Department of Microbiology and Virology, Faculty of Natural Sciences, Comenius University in Bratislava, Ilkovičova 6, 842 15 Bratislava, Slovakia; lucia.cernakova@uniba.sk; 2 Department of Parasitology and Mycology, School of Medicine, Iran University of Medical Sciences, Tehran 1449614535, Iran; roudbari.mr@iums.ac.ir; 3 Centre of Biological Engineering, LIBRO—‘Laboratório de Investigação em Biofilmes Rosário Oliveira’, University of Minho, 4710-057 Braga, Portugal; susana.bras@gmail.com; 4 Microbiology Department, University Hospital “Shefqet Ndroqi”, 1044 Tirana, Albania; stafaj@hotmail.com; 5 LEPABE—Laboratory for Process Engineering, Environment, Biotechnology and Energy, Faculty of Engineering, University of Porto, 4200-465 Porto, Portugal

**Keywords:** *Candida auris*, resistance, antifungal, biofilm, infection, novel therapy

## Abstract

*Candida auris* is a novel and major fungal pathogen that has triggered several outbreaks in the last decade. The few drugs available to treat fungal diseases, the fact that this yeast has a high rate of multidrug resistance and the occurrence of misleading identifications, and the ability of forming biofilms (naturally more resistant to drugs) has made treatments of *C. auris* infections highly difficult. This review intends to quickly illustrate the main issues in *C. auris* identification, available treatments and the associated mechanisms of resistance, and the novel and alternative treatment and drugs (natural and synthetic) that have been recently reported.

## 1. Introduction

In the last decades, fungal infections have been growing faster, presenting a serious threat to the global population, due to the antimicrobial resistance issues, but also the fact that there are much fewer drug classes available than bacterial diseases [1,2,3]. Despite the fact that the first isolate of *Candida auris* was reported more than one decade ago, there is still lack of a global identification strategy available, as well as in developing countries, and there is an urgent need for effective therapy against this multidrug-resistant pathogen [4,5]. Indeed, *C. auris* has been identified in more than 35 countries, most of them documented healthcare-associated person-to-person spread [6]. The high rate of transmission of *C. auris* is accelerated by its aptitude to colonize skin and other body sites, as well as its ability to persist for weeks on abiotic surfaces and equipment [7]. Importantly, whole-genome sequencing (WGS) has allowed the identification of 4 major populations (clades) in which isolates cluster by geography (isolates from Pakistan, India, South Africa, and Venezuela during 2012–2015, and a specimen from Japan). The clades are, thus, from South Asian, East Asian, African, and South American (I to IV, respectively) [8,9]. Indeed, all infections have clustered in these 4 clades; however, recently, a potential fifth clade was identified, separated from the other clades by >200,000 single-nucleotide polymorphisms, in a clinical isolate from a 14-year-old girl in Iran who had never traveled outside the country. The patient was diagnosed with *C. auris* otomycosis and her case was the first known *C. auris* case in Iran [5,10]. Although distanced by thousands of single nucleotide polymorphisms, within each clade, isolates were clonal. Diverse mutations in *ERG11* were related directly to azole resistance in all geographic clades. Actually, WGS was proposed nearly immediately, and recently, independent emergence of unlike clonal populations has emerged on 3 continents [9].

The most worrisome aspects associated with infection caused by *C. auris* can be summarized in three levels: High rate of antifungal drug resistance [9], mode and pace of its easy transmission among healthcare workers and patients in hospitals, and misidentification. This last level is related to difficulties in a correct identification of *C. auris* strains, which are commonly misidentified as other phylogenetically-related pathogens, but also due to inadequacies of common diagnostic tools to precise identification of *C. auris* in many countries that result in misidentification and incorrect therapy, which lead to the acquisition of multiple genetic determinants that confer multidrug resistance [11,12,13,14].

This resilient pathogen survives harsh disinfectants, desiccation, high-saline environments (osmotolerance, >10% NaCl), and high temperatures (thermotolerance, >40 °C).

In addition, it rapidly colonizes abiotic surfaces, immunosuppressed individuals, and causes invasive infections that exact a high toll [7,14,15,16,17,18,19]. Studies have demonstrated that the Hog1-related stress-activated protein kinase (SAPK) signaling pathway has a central role in the *C. auris* reaction to osmotic stress, and it is a crucial virulence factor in several human fungi [1,2]. It is also known that patients can be asymptomatically colonized with *C. auris* [20].

Sources have been found within the patient’s room/bathroom including beds (e.g., bedding materials and bed side trolley), bathroom doors and walls, faucets, floors, sinks, medical equipment and disposable/reusable equipment (e.g., oxygen mask, axillary temperature probes, intravenous pole), and even on mobile phones, and can endure between one and two weeks [1,2,3,4,5,6,7,8,9]. This yeast has the ability to survive for weeks on different moist and dry abiotic surfaces (e.g., plastic and steel) [1,5,6,10,11]. On biotic surfaces, *C. auris* can be found on skin in general, and, in particular, on hands and nares of care providers and healthcare workers [4,9,10,11].

The colonization can spread this yeast to other patients, and colonized patients can progress to invasive and/or superficial infections. It is, thus, extremely important to quickly and correctly identify persons with *C. auris*, in order to contain the spread of *C. auris* [20].

In terms of infection patterns, a recent report showed that there seem to be no significant differences between *C. auris, Candida albicans, Candida glabrata*, or *Candida haemulonii*. The highest in vivo fungal load of the isolates was demonstrated to be detectable in kidney followed by spleen, liver, and lung tested with three different inocula on two different experimental days [1], as similar to previous works [2]. The study indicated no significant difference in the in vivo survival rate between *C. auris* and either *C. albicans* or *C. glabrata*. The tissue fungal burden of *C. auris* was also similar to *C. albicans*. In terms of pathogenicity, there was a comparable pathogenic potential in disseminated infection in immunocompetent hosts, which also emphasized a higher pathogenic potential of *C. auris*, compared to other *Candida* spp. [1,3].

Until now, a dramatic increase of resistance to antifungal drugs has been reported toward *C. auris* in different countries, causing either evasion from the efficient therapeutic options or high frequency of mortality [21,22,23,24]. To date, there is a paucity of data to guide the optimal management of *C. auris*. Based on current ambiguous breakpoints by the CDC, it seems that in the USA about 90, 30, and <5% of isolates have been resistant to fluconazole (Flu), amphotericin B (AmB), and echinocandins, respectively, which has resulted in a low therapeutic success [25]. Echinocandins are currently recommended as first-line therapy for infection in adults and children ≥2 months of age. For neonates and infants <2 months of age, AmB deoxycholate is recommended [26]. In accordance with previous studies, clinical isolates of *C. auris* represented the highest resistance against azoles globally. *C. auris* is intrinsically resistant to Flu and can also be resistant to echinocandin and AmB [11,21,22,27]. Both rapid emergence and pan-resistance to available therapeutic options are worrisome in hospital onset [28].

Additionally, with the ongoing coronavirus disease 2019 (COVID-19) pandemic situation concerning millions of cases worldwide, nosocomial infections including *C. auris* may contribute to worsening of healthcare settings [29,30,31]. Immunodeficient patients are exposed to easy hospital-acquired *Candida* due to its ability to persist on healthcare facilities’ surfaces notwithstanding the decontamination process [32]. Therefore, hospital measures should be strict in screening upon identification of a new *C. auris* case and prevent transmission and spreading by contact-tracing, such as when it was reported in Florida [30], to pre-empt outbreaks of infection. New case reports are increasing regarding *C. auris* infections in patients with COVID-19. Chowdhary (2020) discussed the situation in India, where for four months there was a 60% case-fatality rate of patients with diagnostic coronavirus disease, while two-thirds of them had confirmed *C. auris* infection [29]. Similar news has been reported in America. Four patients in the specialty care unit in Florida with COVID-19 were infected by *C. auris* [30]. Another alarming finding from Mexico revealed that mortality in patients with COVID-19-associated *C.* *auris* bloodstream infection was enormously high—over 83%, even with antifungal therapy [31].

To address the lack of prevalent awareness, inadequacies of conventional diagnostic systems, and therapeutic challenges, this review aimed to investigate the urgent necessity to perform accurate diagnoses and the search for and application of new therapeutic strategies to fight *C. auris*. A structured search of bibliographic databases for peer-reviewed research literature was conducted (e.g., PubMed, WOS), using the words “Candida+auris”, “Candida+auris+resistance”, and “Candida+auris+treatment”.

## 2. *Candida auris* Identification: Methods and Hitches

**Figure 1:**
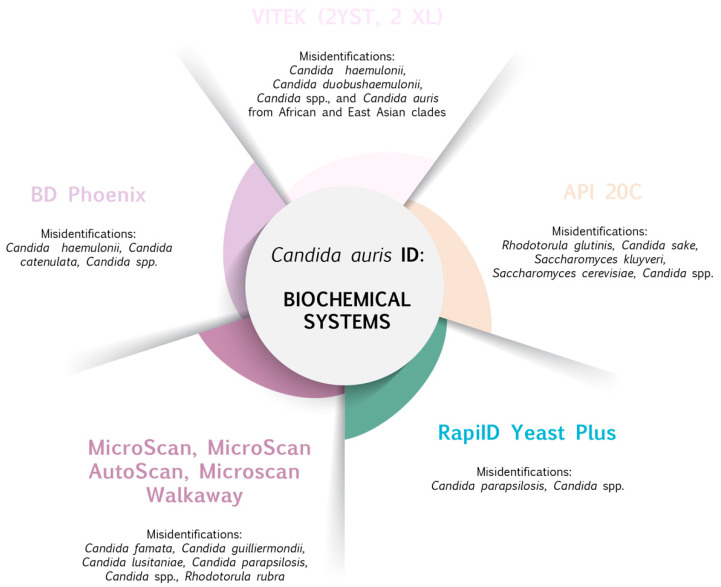
Biochemical-based methods commonly used to identify *C. auris* and species associated with misidentification issues [34,46,47,48].

**Figure 2:**
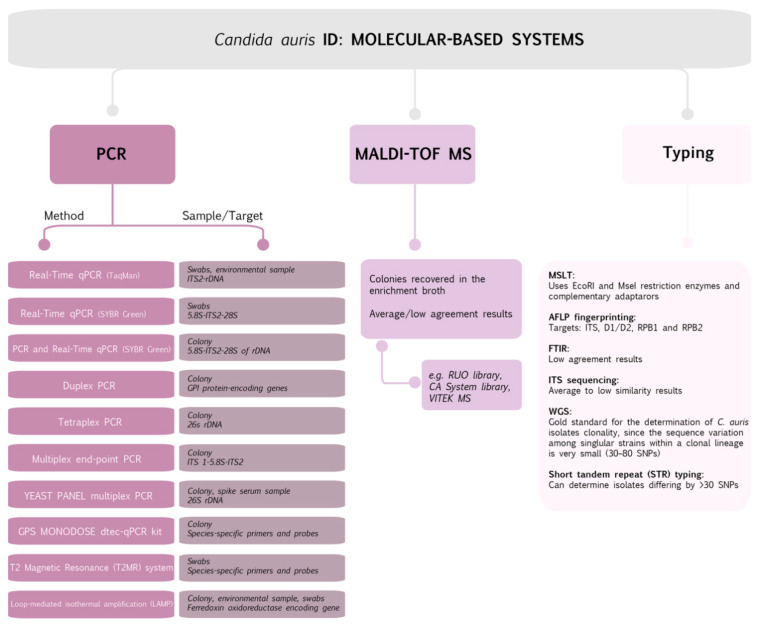
Molecular-based methods commonly used to identify *C. auris* [12,49,50,51,52,53,54,55,56,57,58,59,60,61].

As with other major pathogens, a rapid and correct diagnostic of *C. auris* is essential [4]. Several methods can be used to detect *C. auris* from clinical samples; however, there is still the probability of misdiagnosed species/strains (Figure 1 and Figure 2). For instance, *C. auris* and *C. haemulonii* are closely related and are not readily distinguishable with phenotypic assays [33] and, taking all this into consideration, *C. auris* has been systematically incorrectly identified by phenotypic methods in clinical microbiology laboratories [34]. There are developing countries with a lack of available laboratory technology, where *C. auris* is an unknown pathogen in routine laboratories and most *Candida* isolates are probably misdiagnosed [5]. Importantly, correct identification of *C. auris* is necessary to avoid inappropriate therapy [35]. Cultivation on CHROMagar Candida was described by Kumar et al. (2017). The authors supplemented the medium with Pal’s agar for better differentiation. *C. auris* strains showed confluent growth of white to cream-colored smooth colonies at 37 °C and the *C. haemulonii* complex showed light-pink colonies at 24 h [35,36]. Nevertheless, the new medium CHROMagar™ Candida Plus (CHROMagar, France) allows for a reliable presumptive identification of *C. auris*, as a new specific color—light blue with a blue halo—thus obtaining a sensitivity and specificity of 100% at 36 h of incubation [37]. Another biochemical method is identification by API^®^ 20C, though *C. auris* can be misidentified as *Rhodotorula glutinis, Candida sake,* and *Saccharomyces cerevisiae* [38]. Other methods are, for example, BD-Phoenix and Microscan [39]. The VITEK^®^ 2 system is another tool for identifying *C. auris* strains. Ambaraghassi and colleagues (2019) evaluated a panel of 44 isolates of *Candida* spp. (*C. auris*, *C. haemulonii*, *Candida duobushaemulonii*) using the VITEK^®^ 2 YST ID card [34]. It was also used to investigate the genomic epidemiology of *C. auris* isolated in Singapore [40]. Findings of both groups revealed that VITEK^®^ 2 (version 8.01) yeast identification system has a limited ability to correctly identify *C. auris* and its overall performance probably differs according to the *C. auris* genetic clade [34].

The matrix-assisted laser desorption ionization–time of flight mass spectrometry (MALDI-TOF MS)-based assays offer new perspectives to define the susceptibility pattern of *C. auris*, by species identification, antifungal susceptibility testing (AFST), and typing [41,42,43]. A study by Kwon and others was focused on two MALDI-TOF MS systems, including Biotyper and VITEK^®^ MS, followed by AFST, sequencing of the *ERG11* gene, and genotyping. Using an in vitro diagnostic library of VITEK^®^ MS, 96.7% of the isolates were correctly identified [44]. As another example, isolates collected from blood, urine, ear swab, and groin were confirmed by MALDI-TOF MS and antifungal susceptibility testing was performed using the VITEK^®^ 2 system to detect MICs of six antifungals [45].

It is clear how necessary it is to reliably identify *C. auris* by molecular methods—polymerase chain reaction (PCR) amplification and/or PCR-sequencing of ribosomal DNA (rDNA). The methods are based on sequencing of the D1–D2 region of the 28s rDNA; however, the internal transcribed spacer (ITS) region can also dependably identify *C. auris* [32,39,45,49]. Another option is sequences of 18S rRNA gene and phylogenetic analysis for *C. auris* detection [45]. Sequencing of genetic loci and PCR/qPCR assays have successfully been applied for identification of *C. auris*. The main advantages of PCR identification concerning GPI protein-encoding genes are its low cost, short time, and specificity because of using species-specific primers for *C. auris*, *C. haemulonii, Candida pseudohaemulonii, C. duobushaemulonii*, *Candida lusitaniae*, and *C. albicans.* Importantly, it can be used for detection of the isolate of *C. auris* from spiked blood and serum [4].

## 3. *Candida auris* and Treatment Failure of Common Commercial Antifungal Drugs

*C andida auris* is a multidrug-resistant species associated with high morbidity and mortality in immunocompromised individuals worldwide [12]. As mentioned, the key characteristics of *C. auris* comprise spreading easily in the environment, transiting quickly among hospitalization patients [62,63], as well as being resilient to common disinfectants in the healthcare setting [64]. The lack of availability of microbiological diagnostic methods and subsequent misidentification [65,66] and high levels of antifungal resistance [36,67] makes *C. auris* as a potential virulent species, and globally emerging pathogen, causing challenges to control policies against infection.

The number of antifungal drugs targeting the treatment of *C. auris* infection have been limited. Generally, three main classes of available antifungal including azoles, polyenes, and echinocandins have been used for treatment of infected patients in the clinics [27]. Different countries have reported various levels of resistance to common antifungals used against *C. auris* infections worldwide, as described by Sekyere [68]. In this study, a systematic review and meta-analysis were carried out on 267 *C. auris* clinical isolates in 16 countries in 2019, and the analysis of data displayed that resistance to Flu, AmB, voriconazole (Vcz), and caspofungin was 44.29%, 15.46%, 12.67%, and 3.48%, respectively. Two novel drugs, SCY-078 and VT-1598, are currently in the pipeline [68].

In contrast, the first case report in China investigated that the *C. auris* isolated from bronchoalveolar lavage fluid (BALF) of a hospitalized woman interestingly was sensitive to Flu, AmB, and echinocandins. Additionally, the copper sulfate (CuSO4) also had a potent antifungal effect on *C. auris* [69]. Likewise, *C. auris* isolate as a causative agent of urinary tract infection (UTI) showed sensitivity to AmB and echinocandins, and in the case of refractory and persistence of infections and different combination therapy, flucytosine are recommended as an appropriate alternatives therapy [70].

*C. auris* isolates of patients with candidemia in Russia were susceptible to echinocandins, whereas the high-level of resistance against Flu and AmB was seen in the majority of isolates. More investigation regarding surveillance might be helpful to achieve proper guidelines for the management of candidemia [71].

Response to antifungal agents have also indicated a significant difference between the planktonic and sessile state of *C. auris* growth. *C. auris* enable biofilm structures to form on both medical implementation surfaces in immunocompromised patients and bio-surfaces [50,72] that are strongly associated with the type and phenotypic behavior of the isolates [73]. Additionally, biofilms are capable of triggering the antifungal resistance in this species and developing the persistent infection [74]. Romea et al. found that the minimum biofilm eradication concentration (MBEC) was higher than the minimum inhibitory concentration (MIC) against antifungal drugs. In addition, biofilm was resistant to all classes of antifungals drugs; whereas, it was sensitive to echinocandins and polyenes. Therefore, the biofilm feature seems a fundamental factor to antifungal resistance in *C. auris* [75]. It has been reported that *C. auris* clinical isolates with a mutation in *FKS1* gene were echinocandin resistant, whereas *FKS1* wild-type (WT) isolates were sensitive. The findings suggested micafungin represented the most potent echinocandin against *C. auris*. In addition, all WT isolates showed the Eagle effect (paradoxical growth effect) against the caspofungin susceptibility test performed according to the CLSI microdilution method [76]. However, echinocandins are only available intravenously and are related to increasingly higher rates of resistance by *C. auris.* Thus, a need exists for novel treatments that reveal potent activity against *C. auris*. Recently, therapies focused on echinocandins’ synergism with other antifungal drugs were widely explored, representing a novel possibility for the treatment of *C. auris* infections [77].

## 4. Facing the Challenges to Fight against *Candida auris*: Molecular Resistance Mechanisms

The majority of clinical *C. auris* isolates display resistance to main classes of antifungals (azoles, polyenes, or echinocandins), and there is multi (MDR)- or pan-drug resistance to more than two antifungal classes [13]. Many research groups have studied the molecular mechanisms implicated in resistance development, resulting in limited therapeutic options [13,78]. As previously noted, in order to not fail in treatment of *C. auris* infections, accurate detection and identification of this pathogen is necessary [35,78]. Setting the MICs of antifungals against *C. auris* strains has also been requested, since elevated MICs are severe problem [79]. Importantly, it is equally critical to monitor the antifungal resistance in different geographical areas and implement efficient guidelines for treatment [45].

The antifungal resistance in *C. auris* has been shown to be acquired rather than intrinsic [80]. Currently, the worldwide findings conclude that susceptibility to Flu is the most decreased from *C. auris* isolates, followed by AmB, and 5-fluorocytosine and echinocandins are also not 100% effective [36,81]. WGS studies revealed that *ERG3*, *ERG11*, *FKS1*, *FKS2*, and *FKS3* homologs were found in the *C. auris* genome and these loci share around 80% similarity with *C. albicans* and *C. glabrata* genes [79,82,83]. In general, preliminary genomic studies showed that the targets of several classes of antifungal drugs are conserved in *C. auris*, including the azole target lanosterol 14-α-demethylase (*ERG11*), the echinocandin target 1,3-β-(D)-glucan synthase (*FKS1*), and the flucytosine target uracil phosphoribosyl-transferase (*FUR1*) [36,84].

### 4.1. Mechanisms of Resistance to Azoles

Mechanistically, resistance to azole is acquired through different mechanisms; a number of genes encoding efflux pumps, including ATP-binding cassette (ABC) and overexpressed major facilitator superfamily (MFS) transporters are well known for deliberate major multidrug resistance in *C. auris* [82,85]. The *C. auris* genome contains three genes encoding Mrr1 homologs and two genes encoding Tac1 homologs. Mutations in the zinc cluster transcription factors Mrr1 and Tac1 cause intrinsic Flu resistance of *C. auris* [86]. Deletion of *TAC1b* decreased the resistance to Flu and Vcz in both mutant Flu-resistant strains from clade III and clade IV. It has been shown that the encoded transcription factor is associated with azole resistance in *C. auris* strains from different clades [86]. *CDR1* expression was not or only minimally affected in the mutants, representing that *TAC1b* can confer increased azole resistance by a *CDR1*-independent mechanism. A recent study from Rybak et al. suggested that the Δcdr1 mutation in a resistant isolate was able to increase susceptibility to Flu and itraconazole by 64- and 128-fold, respectively, with notable reductions in MIC also demonstrated in other azoles [87,88]. The function of efflux pumps in azole resistance appears to be predominantly associated with *CDR1*, as analysis of the Δmdr1 mutant showed minimal effects on increasing azole sensitivity [88].

*ERG11* gene mutations are mostly considered to be involved in Flu resistance or higher expression of genes participating in efflux [81,89]. Like other *Candida* species, specific *C. auris ERG11* mutations resulted directly in reduced azole susceptibility [90]. Furthermore, genes encoding efflux pumps may play a role too [33,79,82,83]. Analysis confirmed increased expression of the *CDR1 *orthologous gene belonging to ABC superfamily transporters and supposed involvement in multidrug resistance in *C. auris* together with other transporter genes such as *CDR4*, *CDR6*, and *SNQ2 *[91]. Study of *C. auris* and *C. haemulonii* clinical isolates from 2 hospitals in central Israel revealed that *C. auris* exhibited higher thermotolerance, virulence in a mouse infection model, and ATP-dependent drug efflux activity than *C. haemulonii* [33]. Chawdary et al. found 15 missense mutations when aligned to the *C. albicans* wild-type *ERG11* sequence in *C. auris* azole-resistant in India, and two variants were found in every resistant strain called Y132F or K143R along 5 mutations that were already identified in azole-resistant *C. albicans* [32,36,90]. Isolates with the K143R mutation were resistant to Vcz [32]. Research of AlJindan and colleagues showed that all selected *C. auris* isolates were resistant to Flu and sequencing of *ERG11* revealed the same mutation (F132Y and K143R) in *ERG11* [45]. Similarly, another only two-hour-lasting assay identified mutations Y132F and K143R in *ERG11*, and S639F in *FKS1* HS1, and results were 100% concordant with DNA sequencing results [78]. From the 350 isolates collected in a hospital in India, 90% of *C. auris* were Flu resistant, and 2% and 8% were resistant to echinocandins and AmB, respectively [36]. Interestingly, mutations in *ERG11* were markedly related with geographic clades (i.e., F126T in South Africa, Y132F in Venezuela, and Y132F or K143R in India/Pakistan) [9,79]. These variants have similarly been reported in the *ERG11* gene of *C. auris* in Columbia [90]. Later, overexpression of *ERG11* in resistant isolates of *Candida* spp. overcame the activity of azole drugs to inhibit the synthesis of lanosterol 14α-demethylase. Molecular experiments have shown that resistant *C. auris* expressed the increased level of *ERG11* genes compared to susceptible species; however, until now, this was not examined on susceptible isolates [36].

Furthermore, multidrug resistance in *C. auris* is driven by prior antifungal prescription in hospitals and subsequent antifungal treatment failures [92,93]. However, the resistance/sensitivity profile depends on the specific clades. This is the case of *C. auris* isolates in India, which are almost Flu sensitive, whereas a significant level of Flu resistance has been reported in other geographic areas worldwide [80].

### 4.2. Mechanisms of Resistance to Polyenes

The mechanisms responsible for polyene resistance are poorly understood in *C. auris*. Generally, whole-genome sequencing of resistant isolates has identified four novel nonsynonymous mutations, emphasizing a probable correlation with AmB resistance. The reduction in ergosterol content in the cellular membrane [13,25] is due to overexpression of several mutated *ERG* genes [81]. These mutations included those in genes with homology to the transcription factor *FLO8* gene of *C. albicans* and a membrane transporter [94]. Munoz and colleagues carried out a comparative transcriptional analysis on a resistant isolate and a sensitive isolate after exposure to AmB [84]. Using RNA sequencing (RNA-seq), it was revealed that 106 genes were induced in response to AmB in the resistant isolate. Most notably, genes involved in the ergosterol biosynthesis pathway were highly induced (*ERG1, ERG2, ERG6,* and *ERG13*), which logically showed an association with the maintenance of cell membrane stability [84]. Rhodes et al. screened 27 *C. auris* isolates from the UK for SNPs in *ERG* genes in strains representing reduced sensitivity to AmB. However, variants lighting these differences in drug susceptibility were not found [95].

### 4.3. Mechanisms of Resistance to Echinocandins

The development of resistance to echinocandins is rare, and they are the first-line therapy drugs against *C. auris* infections [13,25]. This resistance is typically associated with hot spot mutations in the *FKS1* gene, which encodes the (1,3)-β-D-glucan synthase enzyme, the target of echinocandins, resulting in lower affinity of the enzyme to the drug [76]. Sequencing of the corresponding hot-spot regions of 38 *C. auris* strains resulted in the discovery that an S639F amino acid substitution was related to pan-echinocandin resistance [36]. Remarkably, this mutation is in the region aligning to the HS1 of *C. albicans FKS1* [95]. Indeed, different mutations have also been revealed in the same location in *C. auris*-resistant isolates including S639Y and S639P, which led to echinocandin resistance in a mouse model in vivo [76]. Identical to other cases of *Candida* spp., changes in the *FKS1* gene leads to caspofungin resistance [81]. On the other side, micafungin-resistant isolates *C. auris* from patients in Kuwait contained the S639F mutation in hot-spot 1 of *FKS1* [32]. Multiple studies have reported the isolation of resistant echinocandins across various geographical regions, with the highest levels of resistance reported in India [76,96].

### 4.4. Mechanisms of Resistance to Flucytosine (5-Fluorocytosine)

Flucytosine, a nucleoside analog, inhibits nucleic acid synthesis. Since this antifungal compound is less used compared to other drugs, the mechanism of resistance is also less understood because fewer studies have been implemented to discover the resistance of *C. auris* to this drug. However, a mutation of *FUR1* was shown to be associated with flucytosine resistance in non-*Candida auris Candida* [97], and mutations in the *FCY2* and *FCY1* genes also seem to be involved in resistance to 5-fluorocytosine [81]. A specific missense mutation of *FUR1* causing F211I amino acid substituted in the *FUR1* gene was demonstrated in resistant *C. auris* that had not previously been reported; therefore, more studies are required to confirm the probable mechanisms responsible for the resistance to flucytosine in the tested *C. auris* strain [95]. Antifungal drugs used for treatment of *C. auris* infection and mechanism of resistance are summarized in Table 1.

## 5. Combination Therapies and Novel Treatments

To fight *C. auris* infections, new therapy methods, drugs, and tools are being tested as an urgent demand. One of the promising approaches seems to be synergistic interactions of compounds with antifungals (Table 2). Published data have also focused on the combination therapy as alternative approaches with antifungal drugs open new insights in the management of a global threat of this multidrug-resistant *C. auris,* as described by Srivastava et al. [98]. In this study, farnesol, a quorum-sensing molecule, suppressed biofilm formation, blocked efflux pumps, and downregulated biofilm and efflux pump-associated genes. Farnesol has been indicated as a promising agent to fight a significant nosocomial multidrug-resistant (MDR) pathogen responsible for causing invasive outbreaks [98]. Similarly, carvacrol, the most active phenolic compound, displayed both antifungal activity with low MIC for clinical isolates of *C. auris* and a synergistic effect in combination with Flu, AmB, caspofungin, and micafungin, reducing the MIC value of these drugs as well. Interestingly, carvacrol strongly inhibited adherence and enzymatic activity, specifically with proteinase as a potential virulence factor developing pathogenesis [99]. Histatin 5 (Hst 5), a salivary cationic peptide, has attracted much attention due to its substantial antifungal activity against multidrug-resistant fungi. Consistently, Hst 5 at a 7.5-μM concentration killed 90% of Flu-resistant clinical isolates of *C. auris*, which was highly sensitive to Hst 5. Hence, Hst 5 signifies wide and potent candidacidal activity, leading it to struggle with MDR *C. auris* strains efficiently [100].

To date, researchers have been encouraged to explore newly synthesized compounds with promising anti-fungal approaches regarding *C. auris* growth in vitro and in vivo. APX001A, a novel agent that inhibits the fungal protein Gwt1 (glycosylphosphatidylinositol-anchored wall transfer protein 1), has been examined against *C. auris*. The findings considered that APX001A has efficient antifungal activity with lower MIC50 and MIC90 than anidulafungin in vitro and also in an immunocompromised murine model with disseminated infection [101].

Moreover, ibrexafungerp (SCY-078), a novel first-in-class antifungal agent targeting glucan synthase, was used to investigate in vitro activity against *C. auris* by applying EUCAST. Ibrexafungerp revealed a promising outcome against *C. auris*, including isolates resistant to echinocandins and/or other agents that were included as comparators [102]. Accordingly, treatment of biofilm with SCY-078 significantly decreased metabolic activity and thickness of sessile cells, indicating that further assessment of this novel bioavailable antifungal is needed [103]. In another study, VT-1598, a tetrazole-based fungal CYP51-specific inhibitor, was assessed in vitro and in vivo against clinical isolates of *C. auris*. VT-1598 significantly reduced the *C. auris* burden in the spleen and brain of the infected mouse in a dose-dependent manner compared to caspofungin. These results propose that VT-1598 may be an effective option for the treatment of invasive infections caused by nosocomial outbreak *C. auris* [104].

Surprisingly, the antifungal activity of crotamine, one of the native polypeptides from the South American rattlesnake, was examined against AmB- and Flu-resistant *C. auris*. This antimicrobial peptide inhibited the growth of *C. auris* at 40–80 µM concentration by using the Clinical and Laboratory Standards Institute (CLSI) microdilution assay in vitro. Therefore, it has been suggested that with regard to the limited efficacy of antifungal drugs, new antimicrobial peptides with a lower chance of inducing resistance compared to chemical antimicrobials have the potential power to fight against multidrug-resistant species [105].

The application of probiotic yeasts as a potential alternative/combination therapy against *Candida* spp., including *C. albicans*, *Candida tropicalis*, *C. glabrata*, *Candida parapsilosis*, *Candida krusei*, and *C. auris*, has been discussed in clinical studies in vitro and in vivo that effectively inhibited *Candida spp.* growth. The only probiotic yeast commercially available is *S. cerevisiae* var*. boulardii*, aggregated to pathogens and able reduce their virulence through inhibiting the adhesion and morphological transition of *Candida* spp., and could be promising therapeutic options [106].

Based on the findings Zhu’s, Jaggavarapu and colleagues announced that administration of micafungin and AmB might have clinical use against *C. auris* and seems to be a suitable combination instead of flucytosine [107,108], which is toxic for some groups of patients with bone marrow problems or for pregnant women [107,109,110]. Currently, the screening is focused on molecules with potential inhibitory effect; for example, sulfamethoxazole showed the most potent in vitro synergistic interactions with Vcz and itraconazole [111]. Likewise, lopinavir is an inhibitor of HIV protease but in combination with azole (mainly itraconazole) exhibited a synergistic effect against *C. auris* [112]. In addition, this agent sensitized *C. albicans*, *C. tropicalis*, *C. krusei*, and *C.* *parapsilosis* to azoles, probably by affecting efflux pump activity [112]. Findings of a similar work indicate synergistic interactions between suloctidil (sulfur-containing aminoalcohol, vasodilator) and Vcz, and between ebselen (a synthetic organoselenium molecule) and anidulafungin [113]. Another case of drug repurposing is aprepitant, an agent helping to prevent nausea and vomiting, and on the other hand to enhance antifungal effects of azoles against *C. auris* by chemosensitizing [114]. Authors reported interference of aprepitant/itraconazole with metal ion homeostasis, which led to losing reactive oxygen species‘ detoxification ability [114].

**Table 2:** Combination therapies used to treat *C. auris* infections.

Combination Therapy	Reference(s)
Micafungin and amphotericin B	[107]
Sulfamethoxazole, Vcz, and itraconazole	[111]
Lopinavir and itraconazole	[112]
Suloctidil (sulfur-containing aminoalcohol, vasodilator) and Vcz	[113]
Ebselen (synthetic organoselenium molecule) and anidulafungin	[113]
Aprepitant and azoles (mainly itraconazole)	[114]
Antimicrobial lock therapy: Caspofungin dissolved in low ionic solutions	[115,116]

Biofilms formed by *Candida* spp. on indwelling catheters are difficult to treat. One of the options to eradicate persistent cells without removal of a catheter is antimicrobial lock therapy [115]. This strategy was selected in the work of Sumiyoshi and colleagues. Caspofungin was dissolved in low ionic solutions and a considerable reduction of candidal (including resistant *C. auris*) was noticed in the polymicrobial biofilms present in the catheter–lock therapy model [116].

New antifungals have been also explored. This is the case of the triazole PC945, which had a higher inhibitory potential than posaconazole, Vcz, and Flu on *C. auris* isolates [117]. Similarly, arylamidine T-2307 causes the collapse of fungal mitochondrial membrane potential and was previously successfully when tested in vitro and in vivo against *Candida* species, also demonstrating activity against *C. auris* [118].

Regarding novel and alternative treatments (Table 3), *C. auris* was found to be surprisingly sensitive to translation inhibition by a class of compounds known as rocaglates (also known as flavaglines, natural products found in Meliaceae plants), which activated a cell death program and, therefore, displayed fungicidal activity against this yeast [119].

Another alternative technique could be irradiation of photoactive dyes by a light source with appropriate wavelength, resulting in reactive oxygen species production, called photodynamic therapy. Methylene blue is a commonly used photosensitizer, and after combination with a light-emitting diode reduced the viability of planktonic as well as biofilm *C. auris* [120]. Finally, a pulsed-xenon ultraviolet mobile device effectively reduced the colony forming unit’s survival of *C. auris* [121]. Potential of UV-C exposure was investigated to constitute room disinfection. The best results for *C. auris* killing were obtained after 30 min of UV-C exposure at 2 m [122]. It is important to notice that *C. auris* has the ability to remain viable on surfaces for at least two weeks [121], which is the main reason that the decontamination processes in hospital surfaces, and on patient medical devices and rooms, is crucial [121,122].

## 6. Conclusions and Future Perspectives

*Candida auris* has recently emerged as a serious and major fungal pathogen. Although it was first identified in Japan about a decade ago, presently, several outbreaks have been claimed in several other countries (e.g., Brazil, United States, India, Mexico, Colombia, and in Europe). The main problem is the multi/pan-drug resistance that this yeast presents, which makes the treatment a higher challenge, with, unfortunately, high rates of treatment failure in all antifungals’ classes. Indeed, the fast emergence of antimicrobial resistance traits pose a significant challenge and reduce treatment options all over the world, which urgently calls for new, broadly effective and cost-efficient therapeutic strategies.

As seen, an early and correct identification of patients colonized with *C. auris* is critical in containing its spread. The elaboration of official guidelines (for instance, from WHO, ECDC, CDC) related to the correct isolation and culture and best methods to avoid misidentification are urgently needed. Additionally, since phenotypic resistance seems to be connected to worse outcomes, this should be taken in perspective with other patient factors (e.g., immunosuppression, previous antibiotherapy). In the occurrence of an undesirable outbreak and having to count the present data on risk factors and transmission mechanisms and the actual pandemic state, contingencies plans should be already prepared in each country, so that all nations could be prepared to act as soon as possible, guiding control measures, to avoid the spread of this pan-resistant pathogen.

Presently, the hope relies on novel and alternative treatments, such as natural compounds (e.g., rocaglates), photodynamic therapy, and novel triazoles (e.g., PC945). Finally, antimicrobial stewardship activities can increase patient results, cut drug adverse effects, and reduce antimicrobial resistance. Collectively, new, precise, and fast diagnostic methods have been established to accelerate effective patient management and progress infection control measures, finally reducing the potential for *C. auris* transmission. A broad attempt involving clinician, laboratory, and healthcare institutes is required to overcome the challenging of preventing transmission of and treatment for *C. auris*.

## Figures and Tables

**Table 1:** Antifungal drugs commonly used against *C. auris* infection and mechanisms of resistance already reported.

Antifungal Drug Class	Mode of Action	Mechanism of Resistance	Reference(s)
Azoles	Inhibit the activity of lanosterol 14-α-demethylase enzyme; prevent converting lanosterol to ergosterol, leading to damaging integrity of cell membrane.	Overexpression of ATP-binding cassette (ABC) and major facilitator superfamily (MFS) transporters. *ERG11* point mutation: Y132F and K143R. Mutation in zinc cluster transcription factors Mrr1 and Tac1.	[9,32,36,45,79,82,85,86,87,88,89,91,92,93]
Polyenes	Bind ergosterol molecules in the cytoplasmic membrane; disturb the permeability of cell membrane by formation of pores, causing oxidative damage.	Induction of genes associated with sterol biosynthetic process including *ERG1*, *ERG2*, *ERG6*, and *ERG13*. SNPs in different genomic loci related to increased resistance.	[81,84,94,95]
Echinocandins	Inhibit β-(1,3)-D-glucan synthase enzyme, leading to defective cell wall formation.	Hot-spot mutation in *FKS1* gene associated with S639Y, S639P, and S639Y regions and *FKS2*.	[32,36,76,81,96]
Flucytosine	Inhibit the nucleic acid synthesis (DNA and RNA) of fungi.	Mutation of *FUR1* gene, specifically missense mutation of *FUR1* causing F211I amino acid substituted in the *FUR1* gene in one flucytosine-resistant isolate. Mutations in the *FCY2*, *FCY1* genes.	[81,95,97]

**Table 3:** Novel and alternative (synthetic and natural) therapies to treat *C. auris* infections.

Therapy	Reference(s)
Triazole PC945	[117]
Arylamidine T-2307	[118]
Rocaglates (flavaglines, from Meliaceae plants)	[119]
Photodynamic therapy (using methylene blue) in combination with light-emitting diode	[120]
Pulsed-xenon ultraviolet mobile	[121]
UV-C exposure (30 min at 2 m)	[122]
